# Dynamin‐related protein 1‐mediated mitochondrial fission contributes to IR‐783‐induced apoptosis in human breast cancer cells

**DOI:** 10.1111/jcmm.13749

**Published:** 2018-07-11

**Authors:** Qin Tang, Wuyi Liu, Qian Zhang, Jingbin Huang, Changpeng Hu, Yali Liu, Qing Wang, Min Zhou, Wenjing Lai, Fangfang Sheng, Guobing Li, Rong Zhang

**Affiliations:** ^1^ Department of Pharmacy The Second Affiliated Hospital of Army Medical University Chongqing China

**Keywords:** apoptosis, breast cancer, Drp1, IR‐783, mitochondrial fission

## Abstract

IR‐783 is a kind of heptamethine cyanine dye that exhibits imaging, cancer targeting and anticancer properties. A previous study reported that its imaging and targeting properties were related to mitochondria. However, the molecular mechanism behind the anticancer activity of IR‐783 has not been well demonstrated. In this study, we showed that IR‐783 inhibits cell viability and induces mitochondrial apoptosis in human breast cancer cells. Exposure of MDA‐MB‐231 cells to IR‐783 resulted in the loss of mitochondrial membrane potential (MMP), adenosine triphosphate (ATP) depletion, mitochondrial permeability transition pore (mPTP) opening and cytochrome c (Cyto C) release. Furthermore, we found that IR‐783 induced dynamin‐related protein 1 (Drp1) translocation from the cytosol to the mitochondria, increased the expression of mitochondrial fission proteins mitochondrial fission factor (MFF) and fission‐1 (Fis1), and decreased the expression of mitochondrial fusion proteins mitofusin1 (Mfn1) and optic atrophy 1 (OPA1). Moreover, knockdown of Drp1 markedly blocked IR‐783‐mediated mitochondrial fission, loss of MMP, ATP depletion, mPTP opening and apoptosis. Our in vivo study confirmed that IR‐783 markedly inhibited tumour growth and induced apoptosis in an MDA‐MB‐231 xenograft model in association with the mitochondrial translocation of Drp1. Taken together, these findings suggest that IR‐783 induces apoptosis in human breast cancer cells by increasing Drp1‐mediated mitochondrial fission. Our study uncovered the molecular mechanism of the anti‐breast cancer effects of IR‐783 and provided novel perspectives for the application of IR‐783 in the treatment of breast cancer.

## 1 INTRODUCTION

Near‐infrared (NIR) imaging is promising for in vivo cancer detection because of its well‐visualized and high tissue penetration depth. NIR optical imaging takes advantage of the wavelength range of approximately 700‐1000 nm, wherein the interference from tissue autofluorescence is minimized.^1^ There are several kinds of NIR in use, including the organic NIR dyes such as BODIPY derivatives, cyanine dye, the inorganic dye‐like gold nanoparticle, and carbon‐based and copper sulphide‐based nanomaterials.^2^ Among various NIR agents, indocyanine green (absorption at ~780 nm and emission at ~820 nm) is the only FDA‐approved NIR dye for clinical applications in angiography, blood flow evaluation and liver function assessment. Moreover, recent studies have shown that indocyanine green is well‐tolerated and can be injected into patients for NIR cancer imaging during cancer surgery.^3^, ^4^ However, indocyanine green is not suitable for diagnostic and therapeutic applications due to the lack of penetration of the emitted light through tissues and the skin and it is without selective antitumour activity.^5^ Thus, it is very important to find a new NIR dye with diagnostic and therapeutic activity that will provide a new possible therapeutic strategy for cancer treatment.

Recently, several reports showed that another kind of NIR, heptamethine cyanine dyes, such as IR‐780 iodide,^6^, ^7^ IR‐783^8^ and MHI‐148^9^ with dual imaging and tumour targeting properties, can be further exploited to improve cancer detection, diagnosis and therapy.^10^ In particular, IR‐783 is a promising imaging reagent for human cancer treatment due to its highly selective cancer targeting activity that can target tumours of the prostate, bladder, pancreas and kidney in vitro and in vivo, and it can reduce the cell viability of cancer cells.^8^, ^10^, ^11^ Moreover, IR‐783 is a low toxicity water‐soluble heptamethine cyanine dye with rapid clearance and is unlikely to be trapped in the reticular and endothelium of the liver or spleen.^12^ Therefore, IR‐783 is a prospective compound that could be further exploited for cancer treatment. However, the exact molecular mechanism by which IR‐783 exerts anticancer effects remains unclear.

It has been reported that the selective antitumour activity of IR‐783 may be mainly related to mitochondria.^10^, ^13^ Yuan et al reported that a strong IR‐783 signal was detected in prostate cancer tissues but not in normal tissues, and their research showed that IR‐783 staining was almost completely colocalized with MitoTracker (a mitochondrial‐selective probe) in cancer cells. Moreover, they also identified that the organic anion transporter polypeptide (OATP), which is highly expressed in cancer cells, mediates the uptake of heptamethine cyanine dyes such as IR‐780,^14^, ^15^ MHI‐148^9^ and IR58 16 into the mitochondria of tumour cells. These results all suggest that the anticancer property of IR‐783 is related to mitochondria.

Mitochondria, crucial double membrane‐bound organelles in cells, play an important role in a diverse range of physiological processes, including cell metabolism, proliferation and differentiation, survival and apoptosis.^17^, ^18^ Mitochondria exist as dynamic networks that maintain the normal shape, structure, quantity and function of mitochondria, and these dynamics are maintained by 2 opposing processes: fission and fusion.^19^ Mitochondrial fission and fusion appear to be essential for cell function and tissue development, and imbalanced fission and fusion always lead to mitochondrial structural changes and dysfunction. Notably, dynamin‐related protein 1 (Drp1) is essential for mitochondrial fission. Drp1, a member of the dynamin family of GTPases, can translocate from the cytosol to the mitochondria and mediates mitochondrial fission, resulting in a loss of mitochondrial membrane potential and apoptosis.^20^ Inhibition of Drp1 blocks mitochondrial fission and cell death.^21^ Recently, a study reported that Drp1 was up‐regulated in certain types of cancers, such as lung and breast cancer.^22^ Moreover, Drp1 is a newly discovered therapeutic target for tumour initiation,^23^ migration^24^ and proliferation.^25^ In 2013, Zhao et al reported that cancer cell migration and invasion were regulated by mitochondrial dynamics.^17^ Thus, targeting Drp1‐dependent mitochondrial dynamics may provide a novel therapeutic strategy for cancer treatment.

In this study, we found that IR‐783 induced breast cancer cell apoptosis by increasing mitochondrial fission. IR‐783 treatment up‐regulated the mitochondrial fission proteins Drp1, mitochondrial fission factor (MFF) and fission‐1 (Fis1), and down‐regulated the fusion proteins mitofusin1 (Mfn1) and optic atrophy 1 (OPA1). Importantly, knockdown of Drp1 attenuated IR‐783‐mediated mitochondrial fission, mitochondrial injury and apoptosis, and inhibition of tumour growth by IR‐783 in an MDA‐MB‐231 xenograft mouse model was also related to the mitochondrial translocation of Drp1 in vivo. Our findings provide a novel mechanistic basis for the application of IR‐783 in the treatment of breast cancer.

## 2 MATERIALS AND METHODS

### 2.1 Chemicals and reagents

The heptamethine cyanine dye IR 783 (2‐[2‐[2‐chloro‐3‐[2‐[1,3‐dihydro‐3,3‐dimethyl‐1‐(4‐sulfobutyl)‐2H‐indol‐ 2‐ylidene]‐ethylidene]‐1‐cyclohexen‐1‐yl]‐ethenyl]‐3,3‐dimethyl‐1‐(4‐sulfobutyl)‐3H‐indolium) was purchased from Sigma‐Aldrich (115970‐66‐6). MitoTracker Red CMXRos (M7512) was purchased from Life Technologies. The following antibodies were used: anticytochrome c (Cyto C, sc‐13156), anti‐OPA1 (sc‐393296), anti‐MFF (sc‐398617), anti‐Mfn1 (sc‐166644) and anti‐Fis1 (SC‐376447) were from Santa Cruz Biotechnology; anti‐Drp1 (611113) was from BD Biosciences; anti‐actin (065M4837V) was from Sigma‐Aldrich; anti‐cleaved‐caspase‐3 (C‐Caspase‐3, 9661), anti‐Cox IV (3E11) and anti‐cleaved‐poly ADP‐ribose polymerase (C‐PARP, 5625) were from Cell Signaling Technology.

### 2.2 Cell culture

The human breast cancer cell line (MDA‐MB‐231) and normal hepatocyte cell line (LO_2_) used in this study were purchased from ATCC (Manassas, VA), cultured in DMEM (Gibco) with 10% foetal bovine serum (TBD Science, TianJin), and incubated at 37°C with 5% CO_2_.

### 2.3 MTT cell viability assay

Cells (5 ×  10^3^ cells/well) were seeded in 96‐well plates. After treatment with IR‐783, 20 μL/well of MTT solution (5 mg/mL, Sigma) was added to the culture medium. After 4 h incubation, the medium was removed carefully and 150 μL of DMSO was added to each well. The plates were detected at a wavelength of 490 nm using a microplate reader (Multiskan Go Thermo Scientific).

### 2.4 Annexin V/PE assays for apoptosis

Cells were harvested and washed twice with cold PBS, then stained with 5 μL of annexin V‐FITC (BD PharMingen, San Diego, CA) and 5 μL of PE (BD PharMingen) in 1 mL binding buffer for 15 min at room temperature in the dark. The apoptotic cells were determined using a Becton‐Dickinson FACScan cytofluorometer (Mansfield, MA).

### 2.5 Detection of mitochondrial membrane potential (MMP) by fluorescence microscope

Cells were seeded in 24‐well plates and cultured overnight. After treatment with IR‐783, cells were stained with JC‐1 dye (Beyotime Institute of Biotechnology, Shanghai, China, C2006) for 20 minutes at 37°C. Afterwards, cells were washed twice with JC‐1 buffer solution. JC‐1 aggregates (red fluorescence) and JC‐1 monomers (green fluorescence) were observed immediately by fluorescence microscopy (CKX31 OLYMPUS, Japan).

### 2.6 Detection of MMP by fluorescence microplate reader

Mitochondrial membrane potential (MMP) was measured by the uptake of the fluorescent dye rhodamine‐123. Briefly, cells were washed twice with PBS, stained with rhodamine‐123 (1 μmol/L) in serum‐free medium for 30 minutes at 37°C. After that, the medium was removed and fresh medium was added for detection. The fluorescence intensity was measured by a fluorescence microplate reader (Thermo Varioskan™ LUX) at the excitation wavelength of 507 nm and the emission wavelength of 529 nm, respectively. The results are expressed as a percentage of the control, which was set at 100%.

### 2.7 Measurement of cellular content of adenosine triphosphate (ATP)

To detect cellular ATP production, the ATP Assay Kit (Beyotime Institute of Biotechnology, Shanghai, China, S0026) was used. After drug treatment, cells were collected and washed twice with PBS. Then, we added ATP Cell Lysis solution to dissolve the cells, followed by centrifugation at 12 000 *g* for 10 minutes at 4°C, and the supernatant was removed and mixed with dilution buffer containing luciferase. The luminescence value was detected using a microplate reader (Thermo Varioskan™ LUX) according to the manufacturer's instructions. A fresh standard curve was prepared each time and the ATP content was calculated using this curve. The results are expressed as a percentage of the control, which was set at 100%.

### 2.8 Measurement of mitochondrial permeability transition pore (mPTP) opening

mPTP opening analysis was performed as previously described.^26^ Briefly, after drug treatment, the cells were washed twice with PBS and stained with calcein‐acetoxymethyl ester (calcein‐AM) and CoCl_2_ in serum‐free medium for 15 minutes at 37°C. After that, the medium was removed and fresh medium was added for detection. The extra‐mitochondrial Ca^2+^ concentration was measured by a fluorescence microplate reader (Thermo Varioskan**™** LUX) at the excitation wavelength of 488 nm and the emission wavelength of 525 nm. The results are expressed as a percentage of the control, which was set at 100%.

### 2.9 Western Blot Analysis

Cells and tumour tissues were harvested and lysed in cell lysis solution (Beyotime Institute of Biotechnology, Shanghai, China, P0013) with 10% PMSF. The mitochondria of the cells and tumour tissues were extracted as described by the manufacturer (Beyotime Institute of Biotechnology, Shanghai, China, C3601). The protein concentration was quantified using a BCA protein assay kit (Beyotime Institute of Biotechnology, Shanghai, China, P0010). Equal quantities of protein (generally 15, 30 or 60 μg) were resolved by SDS‐PAGE in sample loading buffer. Samples were separated on 8‐12% gels and then transferred to 0.22 μm polyvinylidene difluoride membranes (Millipore). The membrane was then blocked with 5% (w/v) non‐fat milk in TBS and 0.1% Tween 20 (TBS/T). After washing with TBS/T, the PVDF membrane was incubated with anti‐C‐Caspase‐3 (diluted 1:500), anti‐PARP (diluted 1:500), anti‐Drp1 (diluted 1:500), anti‐Cox IV (diluted 1:500), anti‐actin (diluted 1:2000), anti‐Cyto C (diluted 1:1,000), anti‐OPA1 (diluted 1:500), anti‐Fis1 (diluted 1:500), anti‐MFF (diluted 1:500), and anti‐Mfn1 (1:500) primary antibodies overnight at 4°C, followed by incubation with horse radish peroxidase‐conjugated secondary antibody for 1 hour at room temperature. Proteins were visualized with a luminol substrate solution.

### 2.10 Plasmids and establishment of stable cell lines

A Drp1 shRNA (shDrp1, target sequences: 5′CCGG CGGTGGTGCTAGAATTTGTTA CTCGAG TAACAAATTCTAGCACCACCG TTTTTG3′) plasmid was purchased from Sigma. Plasmids were transfected along with lentiviral packaging vectors such as pLP1, pLP2, and pLP/VSVG (Invitrogen, K4975) into 293FT cells by Lipofectamine 3000 (Invitrogen, L3000015) according to the manufacturer's protocols. The supernatant containing the lentivirus was harvested 48 hours later and was used to infect MDA‐MB‐231 cells. Cells were subsequently selected with 10 μg/mL puromycin (Sigma, P9620) to establish stable cell lines.

### 2.11 Transmission electron microscopy assay

For electron microscopy, cells were fixed in 2.5% glutaraldehyde at 4°C for 24 hours, fixed in 2% osmium tetroxide at 4°C for 2 hours, dehydrated with a series of ethanol and embedded in Epon Ultrathin. Subsequently, sections were prepared using a microtome (UC7, Leica, Germany) and stained with uranyl acetate and lead citrate. Mitochondria were examined with a Tecnai 10 transmission electron microscope (Philips, Netherlands).

### 2.12 Immunofluorescence

MDA‐MB‐231 cells were plated on coverslips and cultured in 24‐well plates for 24 hours, and after drug treatment, the cells were stained with 100 nmol/L MitoTracker Red CMXRos for 30 minutes, then washed with culture medium 5 times. Then, the cells were fixed, permeabilized and blocked with 5% milk. Immunostaining was performed using antibodies including anti‐Drp1 (1:50), followed by incubation with a secondary antibody Alexa Fluor 488 goat antimouse (A1101, Molecular Probes). A laser‐scanning confocal microscope (LSM780NLO, Zeiss, Germany) with a 63× oil objective was used for cell imaging. Mitochondrial length was measured by an investigator blinded to the samples performed with the polygon tool of Imaris 3D software. The colocalization of Drp1 and mitochondria was analysed using ImageJ software.

### 2.13 Animal experiments

All animal studies were approved by the Army Medical University Institutional Animal Care and Use Committee. Female nude mice (5 weeks old) were purchased from Vital River Laboratories (VRL, Beijing, China). MDA‐MB‐231 cells were mixed with Matrigel (1:1, Corning Biosciences, 356234) and injected subcutaneously into the right hind legs of nude mice. Mice were randomized into 2 groups (n = 5). IR‐783 (20 mg/kg) or an equal volume of vehicle was administered daily by intravenous injection starting 7 days after tumour inoculation. Tumour growth and mice bodyweights were measured every week, and tumour volume was calculated as (length × width^2^)/2. After 4 weeks of treatment, mice were killed by cervical dislocation, and tumour tissues were harvested and fixed in 4% paraformaldehyde or frozen at −80°C. Histological, immunohistochemical and immunofluorescence analyses were performed as described.^27^ TdT‐mediated dUTP nick‐end labelling (TUNEL) analysis was used to detect the dead cells in the tissue samples with *In Stitu Cell Death Detection kit*(Roche, Mannheim, Germany) according to the manufacturer's instructions. Tumour tissues from each group were lysed and subjected to western blot analysis.

### 2.14 Statistical analysis

All data are shown as the mean ± SD. Comparisons between individual groups were made with a 2‐tailed Student's *t*‐test. Comparisons between multiple groups were made with analysis of variance, and * *P *<* *.05, **P <* *.01 or ***P <* *.001 were considered significant.

## 3 RESULTS

### 3.1 IR‐783 inhibits cell viability and induces apoptosis in human breast cancer cells

**Figure 1:**
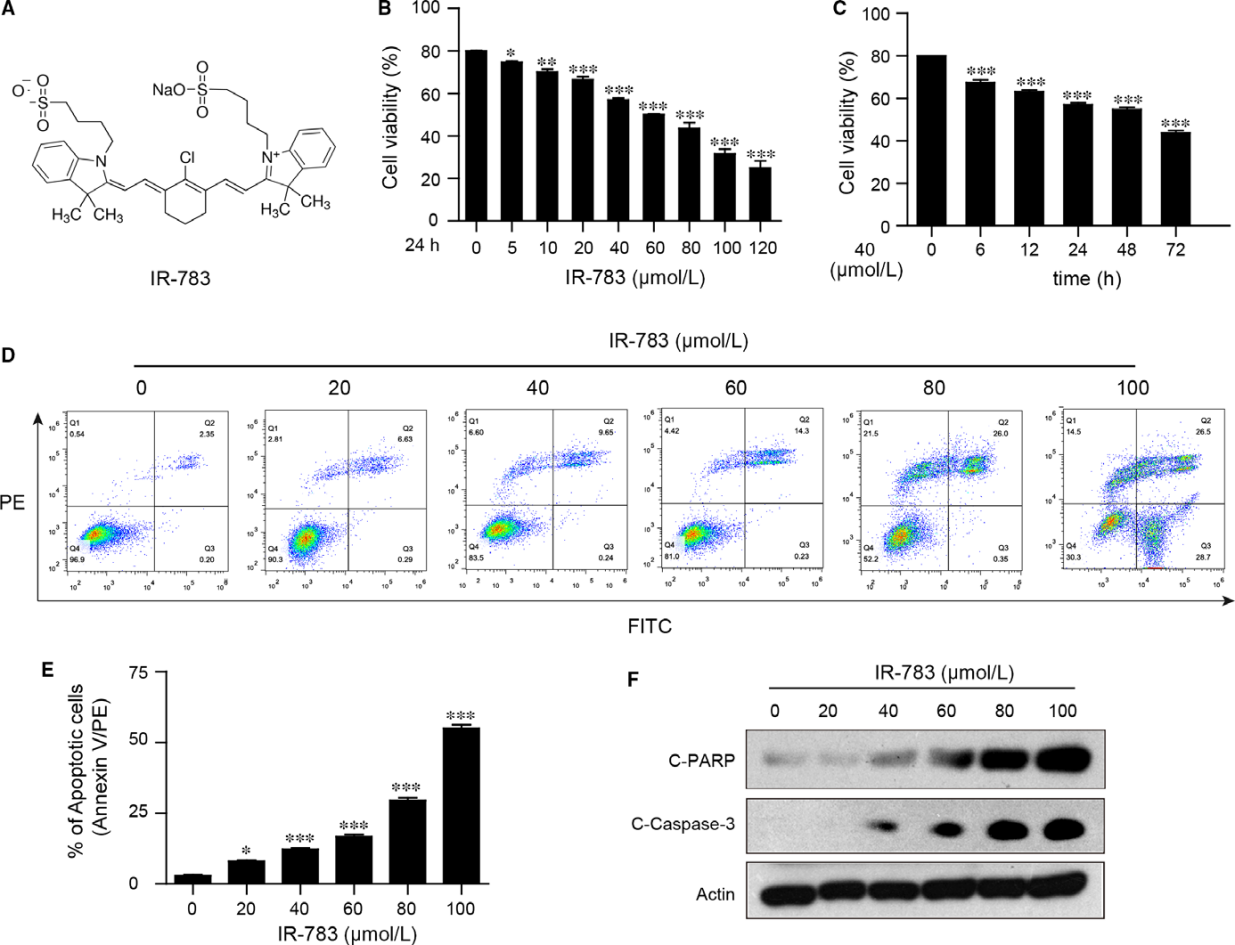
IR‐783 inhibits cell viability and induces apoptosis in human breast cancer cells. A, The structural formula of IR‐783. B and C, Human breast cancer MDA‐MB‐231 cells were treated with various IR‐783 concentrations for 24 h or with 40 μmol/L IR‐783 for different time intervals and then cell viability was detected using MTT assays. The results were counted in 3 independent experiments (n = 3). Data are expressed as a percentage of the control, which was set at 100%. D and E, MDA‐MB‐231 cells were treated with various concentrations of IR‐783 for 24 h. The percentage of apoptotic cells was measured by flow cytometry using annexin V‐FITC/PE staining. The results were counted in 3 independent experiments. F, MDA‐MB‐231 cells were exposed to various concentrations of IR‐783 for 24 h and the expression of apoptosis‐related proteins cleaved‐ poly ADP‐ribose polymerase (C‐PARP) and cleaved‐caspase 3 (C‐Caspase‐3) were detected by western blot analysis. Actin was used as the loading control (*P <* *.05, **P <* *.01, ***P <* *.001 compared to control cells)

We first detected the effects of IR‐783 on cell viability in human breast cancer MDA‐MB‐231 cells at different doses and different time intervals performed with MTT assays. IR‐783 treatment decreased the cell viability in a dose‐ and time‐dependent manner (Figure 1B,C). We also investigated whether IR‐783 inhibited cell viability in human normal hepatocyte LO_2_ cells. In contrast to the breast cancer cell line, IR‐783 treatment had no apparent effects on cell viability of LO_2_ cells even at a concentration of 120 μmol/L (Figure S1). Flow cytometry analysis revealed that exposure of MDA‐MB‐231 cells to IR‐783 for 24 hours resulted in a significant increase in apoptosis (Figure 1D,E). Western blot analysis revealed that IR‐783 increased C‐Caspase‐3 in a dose‐dependent fashion and increased PARP cleavage (Figure 1F). Taken together, these findings suggest that IR‐783 selectively inhibits cell viability and induces apoptosis in human breast cancer cells but not in normal hepatocytes.

### 3.2 IR‐783 induces loss of MMP, ATP depletion, mPTP opening, and Cyto C release

**Figure 2:**
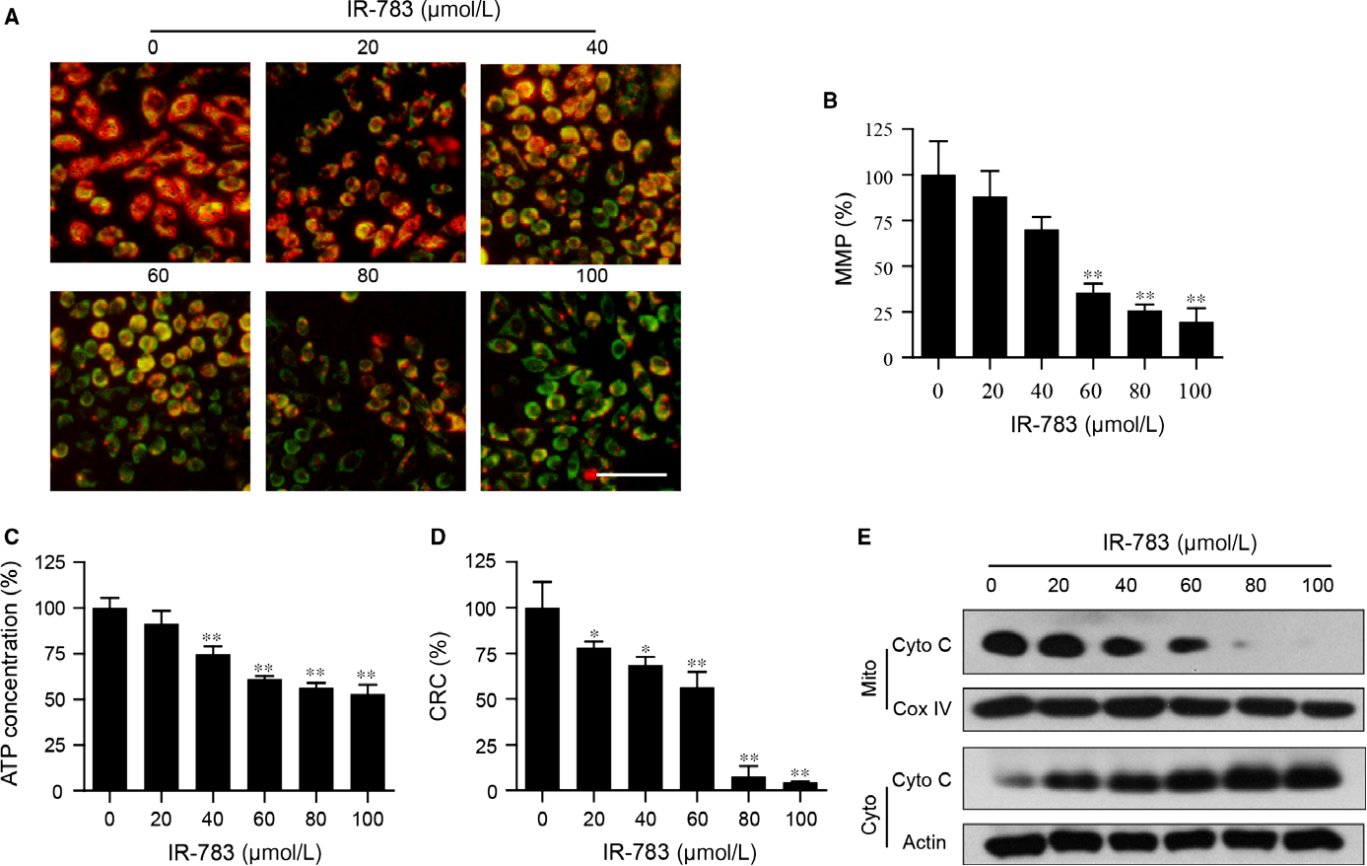
IR‐783 induces mitochondrial injury in MDA‐MB‐231 cells. A, MDA‐MB‐231 cells were treated with various concentrations of IR‐783 for 24 h. The mitochondrial membrane potential (MMP) was measured by JC‐1 staining and detected by a fluorescence microscope. Scale bars: 80 μm. B, MMP was measured by rhodamine‐123 staining and analysed by a fluorescence microplate reader. C, Measurement of intracellular content of ATP by a Luminometer Microplate reader. D, The opening of mitochondrial permeability transition pores (mPTP) was analysed with calcein‐AM+CoCl_2_ staining and detected by a fluorescence microplate reader. The calcium retention capacity (CRC) in contrast to the control group is an index of the opening of mPTP. E, Mitochondrial (Mito) and cytosolic (Cyto) fractions were prepared and subjected to western blot analysis using antibodies against cytochrome c (Cyto C). Actin (cytosolic fraction) and Cox IV (mitochondrial fraction) were used as the loading controls. Values represent the mean±SD for 3 separate experiments. Data are expressed as a percentage of the control, which was set at 100% (*P <* *.05, **P <* *.01 compared to the control group)

Researchers have found that the NIR heptamethine cyanine dyes, such as IR‐780,^6^ IR‐783,^8^, ^28^ IR‐808 and MHI‐148,^8^ show preferential accumulation and retention in different types of cancer cells, with increased uptake in the mitochondria. Mitochondria are recognized as essential organelles for cell homeostasis. MMP depolarization is an important step in mitochondrial pathways during physiological cell death. We first investigated the effects of IR‐783 on MMP by JC‐1 and rhodamine‐123 staining. Fluorescence microscopy results revealed that exposure of cells to IR‐783 resulted in a markedly increased shift of JC‐1 aggregates (red) to JC‐1 monomers (green) (Figure 2A). Microplate reader results showed that treatment with IR‐783 resulted in a significantly decreased fluorescence intensity of rhodamine‐123 in a dose‐dependent manner (Figure 2B). These results suggest that IR‐783 induces a collapse of the MMP in MDA‐MB‐231 cells.

Adenosine triphosphate is an energy molecule that plays an important role in physiology and pathology. The synthesis of ATP occurs via the oxidative phosphorylation of glucose or via the canonical mitochondrial oxidation pathway from ATP synthetase, and thus cellular metabolism is maintained in a tight energy homeostasis through regulation of ATP levels.^29^ Cancer cells have been shown to have higher ATP levels than normal cells in vitro, and decreasing ATP means the cancer cells are entering conditions of apoptosis or necrosis.^30^ As shown in Figure 2C, treating MAD‐MA‐231 cells with IR‐783 significantly decreased the intracellular level of ATP.

Mitochondrial permeability transition pore (mPTP) opening was determined by calcium retention capacity (CRC) and results in the activation of mitochondrial apoptosis in cancer cells.^31^, ^32^ Calcein‐AM, a colourless and nonfluorescent esterase substrate, is highly liposoluble and can enter into living cell membranes easily and then form a very polar green fluorescent material, calcein.^33^ The fluorescence from cytosolic calcein is quenched by the addition of CoCl_2_, while the fluorescence from the mitochondrial calcein is maintained, and a fluorescence detector can detect the fluorescence intensity and estimate the opening degree of mPTP pores. In our results, IR‐783 treatment increased mPTP opening in MDA‐MB‐231 cells as shown by the decreased CRC (Figure 2D). Western blot analysis showed that treatment with IR‐783 resulted in increased release of Cyto C from the mitochondria to the cytosol (Figure 2E). Altogether, these data demonstrate that IR‐783 induces mPTP opening‐dependent mitochondrial injury.

### 3.3 IR‐783 causes mitochondrial fission in MDA‐MB‐231 cells

**Figure 3:**
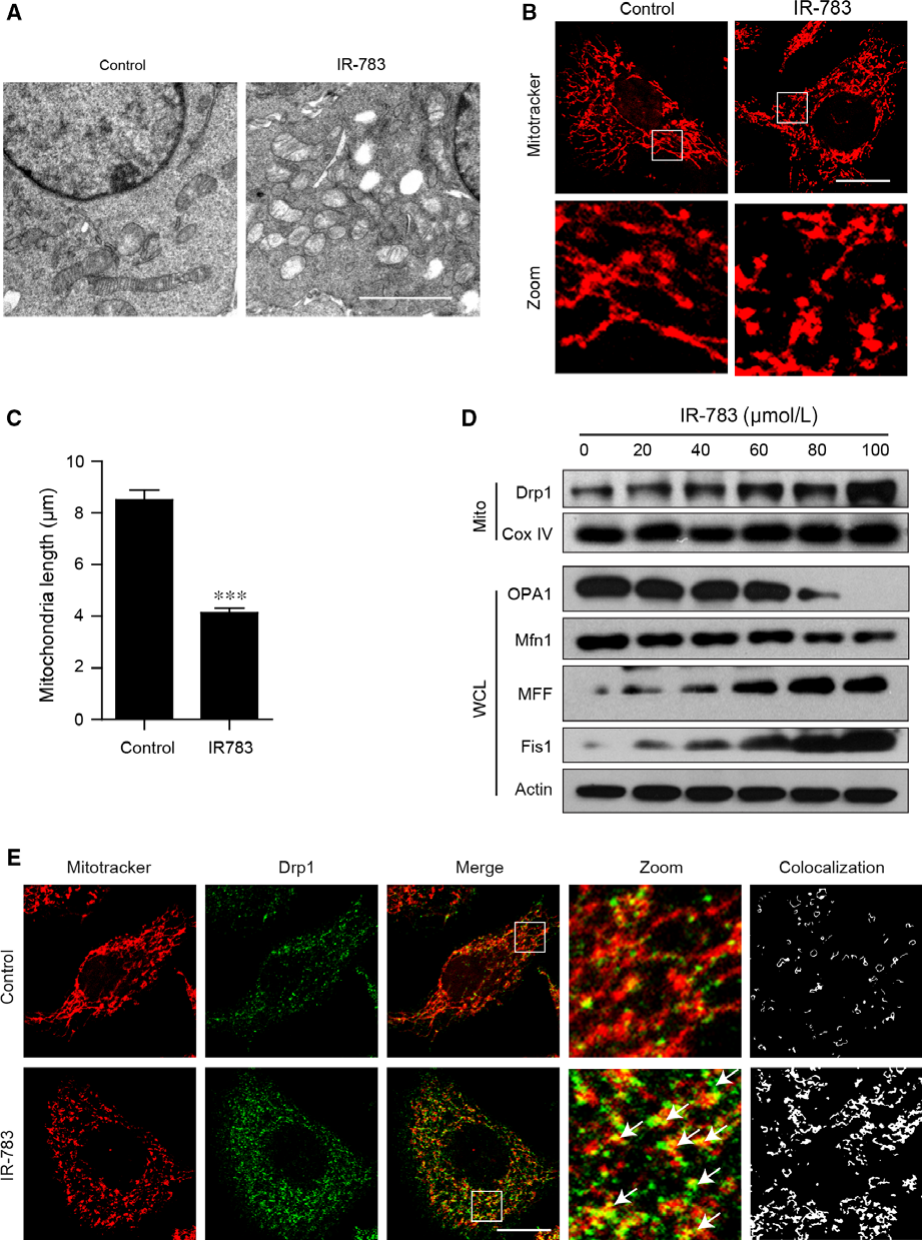
IR‐783 causes mitochondrial fission in MDA‐MB‐231 cells. A, MDA‐MB‐231 cells were treated with 40 μM IR‐783 for 24 h and mitochondria were imaged by transmission electron microscope. Scale bars: 2 μm. B, MDA‐MB‐231 cells were exposed to 40 μmol/L IR‐783 for 24 h. Mitochondria were then stained with MitoTracker Red CMXRos (red) and observed under a confocal microscope. Scale bars: 20 μm. C, Average mitochondrial length was counted in 30 cells. Error bars represent the mean ± SD, ***P <* *.001. D, MDA‐MB‐231 cells were treated with IR‐783 with various concentrations as indicated, and mitochondrial fractions and whole cell lysates were subjected to western blot analysis using anti‐Drp1, OPA1, Mfn1, MFF and Fis1 antibodies. Cox IV (mitochondrial fraction) and actin (whole cell lysates) were used as the loading controls. E, MDA‐MB‐231 cells were treated with 40 μmol/L IR‐783 for 24 h and then the mitochondria were stained with MitoTracker Red CMXRos (red) after immunostaining with Drp1 (Alexa Fluor 488, green) and cells were visualized using confocal microscopy. The colocalization of Drp1 and mitochondria were analysed by ImageJ software. Scale bars: 20 μm

Increasing evidence indicates that mitochondrial fission promotes the initiation of mitochondrial apoptosis.^34^, ^35^ Our transmission electron microscopy assay revealed that mitochondria presented as elongated filamentous structures in control cells, but exhibited small and punctate mitochondria in IR‐783‐treated cells (Figure 3A). In the meantime, we examined the effects of IR‐783 on mitochondria by staining MDA‐MB‐231 cells with MitoTracker Red CMXRos. Exposure of cells to IR‐783 resulted in significantly increased mitochondrial fission and markedly decreased average mitochondria length (Figure 3B,C). Our transmission electron microscope and confocal microscope assay indicated that IR‐783 increased mitochondrial fragmentation. Researchers have previously demonstrated that Drp1 effectively influences the mitochondrial fission process in cells by translocation of Drp1 to the mitochondrial outer membrane.^20^, ^36^, ^37^ To further confirm these results in our experiment, western blot analysis showed that treatment of cells with IR‐783 significantly increased the levels of Drp1 in mitochondria in a dose‐dependent manner, decreased the expression of mitochondria fusion regulators Mfn1 and OPA1, and increased the expression of mitochondria fission regulators MFF and Fis1 in the whole cell lysate (Figure 3D). Immunofluorescence assays were used to further detect the sub‐cellular localization of Drp1 and mitochondria. The results showed that the colocalization of mitochondria and Drp1 was markedly increased after IR‐783 treatment (Figure 3E). These findings suggest that IR‐783 induces mitochondrial fission in MDA‐MB‐231 cells.

### 3.4 Knockdown of Drp1 blocked IR‐783‐induced mitochondrial fission and mitochondrial apoptosis

**Figure 4:**
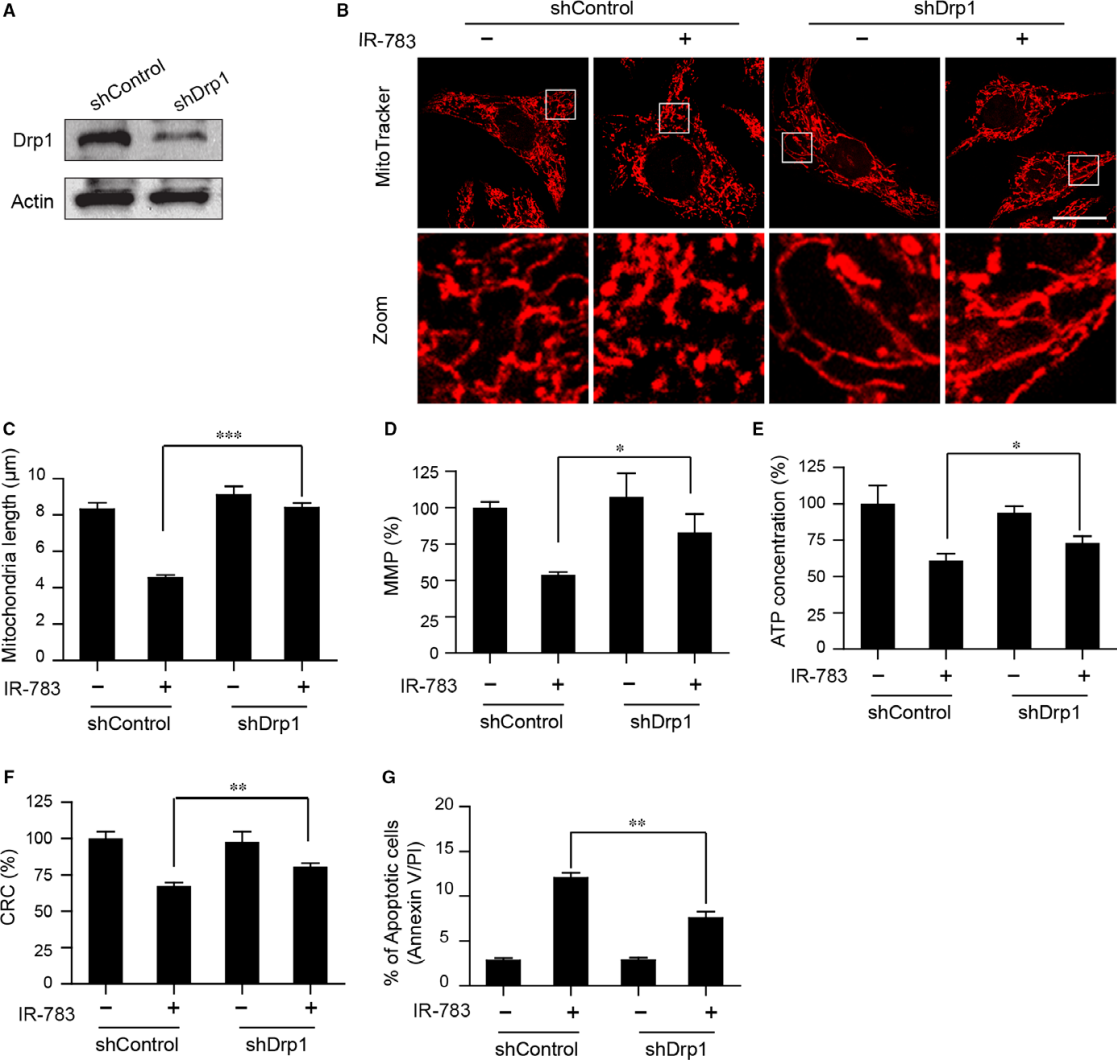
Knockdown of Drp1 blocked IR‐783‐induced mitochondrial fission, loss of MMP, ATP depletion, mPTP opening and apoptosis. A, MDA‐MB‐231 cells stably expressing non‐target shRNA (shCon) or Drp1 shRNA (shDrp1) were lysed and analysed by western blot. Actin was used as the loading control. B, shCon and shDrp1 cells were treated with or without 40 μmol/L IR‐783 for 24 h. Mitochondria were then stained using MitoTracker Red CMXRos (red) and observed under a confocal microscope. Scale bars: 20 μm. C, Average mitochondrial length was counted in 30 cells. D, shCon and shDrp1 cells were treated with or without 40 μmol/L IR‐783 for 24 h then the cells were stained with rhodamine‐123. The MMP was measured by fluorescence microplate. E, Measurement of intracellular content of ATP by Luminometer Microplate reader. F, Cells were stained with calcein‐AM+CoCl_2_ and tested by fluorescence microplate. The calcium retention capacity (CRC) contrast to control group is an index of the opening of mPTP. G, Cells were stained with annexin V‐FITC/PE and the percentage of apoptotic cells was measured by flow cytometry. The results were counted in 3 independent experiments (n = 3). Error bars represent the mean ± SD (*P <* *.05, **P <* *.01, ***P <* *.001)

To prove the important role of Drp1‐regulated mitochondrial fission in the process of IR‐783 induced MDA‐MB‐231 cell death, a lentiviral transduction approach was used to stably knockdown Drp1 expression (Figure 4A). We first evaluated the effects of Drp1 knockdown on mitochondrial morphology. Immunofluorescence microscopic studies showed that knockdown of Drp1 significantly increased the average length of mitochondria and blocked the mitochondrial fission induced by IR‐783 treatment (Figure 4B,C). Mitochondrial fission is considered to be universally associated with the initiation of apoptosis through the mitochondrial apoptotic pathway.^38^, ^39^ We then investigated whether knockdown of Drp1 could affect IR‐783 induced mitochondrial injury and apoptosis. Our results found that knockdown of Drp1 significantly abrogated IR‐783‐mediated loss of MMP, ATP depletion and mPTP opening (Figure 4D,E,F). Meanwhile, flow cytometry analysis revealed that knockdown of Drp1 significantly abrogated IR‐783‐mediated apoptosis (Figure 4G).

### 3.5 IR‐783 inhibited tumour growth by induction of mitochondrial translocation of Drp1 in an MDA‐MB‐231 xenograft mouse model

**Figure 5:**
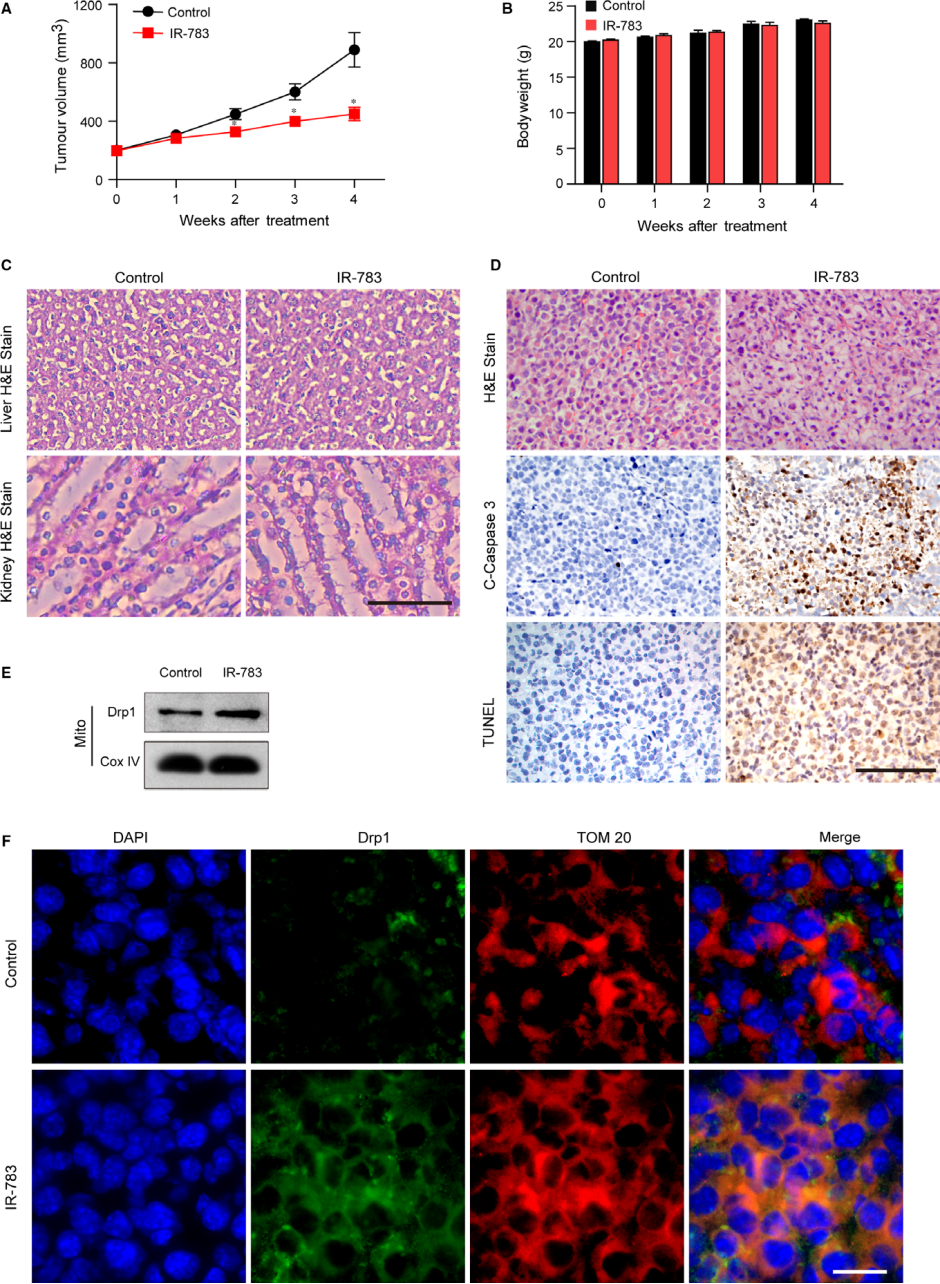
IR‐783 inhibited tumour growth in vivo by induction of the mitochondrial translocation of Drp1. A, Tumour volumes in xenograft mice were measured every week in the control and IR‐783 group *P <* *.05. B, The bodyweight of mice after 4 weeks of IR‐783 treatment. C, Representative liver and kidney tissues were sectioned and subjected to haematoxylin‐eosin (H&E) staining. Scale bars: 100 μm. D, Representative tumour tissues were sectioned and subjected to H&E, TUNEL analysis and immunohistochemical staining for C‐Caspase‐3. Scale bars: 100 μm. E, The mitochondrial fractions of representative tumour tissues were prepared and subjected to western blot analysis using an anti‐Drp1 antibody. Cox IV was used as the loading control for mitochondrial fractions. F, The representative tumour tissues were sectioned and subjected to immunofluorescence using the anti‐Drp1 (green) and anti‐TOM20 (red, a mitochondrial marker) antibody. Scale bars: 20 μm

To determine whether IR‐783 exhibits antitumour activity in vivo, an MDA‐MB‐231 xenograft mouse model was established and received intravenous injections of either vehicle or IR‐783 (20 mg/kg) for 4 weeks. Treatment with IR‐783 resulted in a significant inhibition of tumour growth after 3 weeks of drug exposure compared with the control group animals (*P <* *.05). However, there were no statistically significant changes in bodyweight between the IR‐783‐treated mice and control animals (Figure 5B). Moreover, the representative liver and kidney tissues were sectioned and subjected to haematoxylin and eosin (H&E) staining. The results showed that there were no morphological differences between the IR‐783‐treated mice and control animals, indicating that IR‐783 has a low liver and kidney toxicity (Figure 5C). To further analyse the mechanism whereby IR‐783 inhibited tumour growth, the representative tumour tissues were sectioned and subjected to H&E staining for tissue morphology, TUNEL analysis and immunohistochemistry staining of C‐Caspase‐3 for cell death. As shown in Figure 5D, IR‐783 treatment changed the morphology of the tumours as indicated by signs of necrosis, inflammatory cell infiltration, and fibrosis. Exposure of mice to IR‐783 resulted in an increase in number of TUNEL‐positive cells (dark brown colour) and immunoreactivity for C‐Caspase‐3 (dark brown colour) in the tumour sections, which is indicative of apoptosis.

Furthermore, to evaluate whether mitochondrial fission could be involved in antitumour activity mediated by IR‐783 in vivo*,* representative tumour tissues of each group were homogenized and mitochondria were isolated to investigate mitochondrial translocation of Drp1 by western blot using an anti‐Drp1 antibody. The results showed that treatment with IR‐783 increased the expression of Drp1 in the mitochondrial fractions (Figure 5E). At the same time, representative tumour tissues were sectioned and subjected to immunofluorescence and the results revealed that treatment with IR‐783 increased the colocalization of Drp1 and TOM20 (a marker of mitochondria) (Figure 5F). Taken together, these findings indicated that IR‐783 inhibited tumour growth in vivo by induction of mitochondrial translocation of Drp1.

## 4 DISCUSSION

In this study, we reported that the heptamethine cyanine dye of IR‐783 induced apoptosis in breast cancer cells in vitro and in vivo. Previous studies have found that IR‐783 can display dual imaging and targeting abilities in cancers such as prostate, bladder, pancreatic and kidney cancer and reduce the viability of cancer cells, indicating that IR‐783 is a prospective compound that could be further exploited for cancer treatment.^8^, ^10^, ^11^ Therefore, it is important to reveal the exact molecular mechanism behind the anticancer effects of IR‐783.

Mitochondria are important organelles in eukaryotic cells that exert vital and lethal effects on many biochemical functions, including cell metabolism, growth, differentiation, survival and programmed cell death.^18^ Owing to its roles in the regulation of fundamental cellular functions, it is not surprising that mitochondria have been implicated in multiple aspects of tumour processes.^17^, ^38^ MMP, which reflects mitochondrial functional status, is thought to maintain the respiratory chain to generate adenosine triphosphate, and a decrease in MMP accelerates cellular depletion of ATP, followed by the mPTP opening with subsequent Cyto C release. mPTP opening has been implicated as the determinant cell death pathway in cancer.^32^, ^40^, ^41^ It has been previously reported that the location of IR‐783 was almost completely congruent with MitoTracker (a mitochondrial‐selective probe) in prostate cancer cells, and the cancer‐specific uptake of these organic dyes occurs primarily via OATP1B3. Our results are consistent with these prior findings that IR‐783 induces mitochondrial‐dependent apoptosis based on the following evidence. First, IR‐783 induced apoptosis, Caspase‐3 activation and PARP cleavage in human breast cancer cells. Second, IR‐783 induced loss of MMP, ATP depletion, mPTP opening and Cyto C release in breast cancer cells. Third, IR‐783 significantly inhibited tumour growth at a dosage of 20 mg/kg in vivo and increased the expression of the apoptotic protein C‐Caspase‐3 in tumour tissues.

Mitochondrial fission and fusion appear to be essential for cell function and tissue development.^42^ Changes in mitochondrial morphology are tightly regulated by the balance of fusion and fission processes.^19^ Mitochondrial fission is considered to be universally associated with the initiation of the mitochondrial apoptotic pathway. Notably, Drp1, a highly conserved dynamin‐related cytoplasm GTPase, is essential for mitochondrial fission. After stimulation by fission signals, Drp1 migrates to mitochondria and mediates mitochondrial fission. Fis1 and MFF, which are outer membrane‐anchored proteins of Drp1, were proposed to act in the recruitment and association of Drp1 with mitochondria and are considered to be limiting factors in the fission process.^43^, ^44^ Conversely, the outer membrane Mfn1 and Mfn2 and the inner membrane OPA1 mediate mitochondrial fusion. Mitochondrial fission promotes mitochondrial membrane depolarization, ATP depletion, Cyto C release and cell apoptosis. Our study demonstrated that IR‐783 induces Drp1‐dependent mitochondrial fission in MDA‐MB‐231 cells, based on the following evidence: (i) our transmission electron microscopy and immunofluorescence assay revealed that IR‐783 treatment resulted in mitochondrial fragmentation and significant decreases in the average length of mitochondria. (ii) the western blot results were also consistent with the results of mitochondrial morphology. Indeed, levels of the mitochondrial fusion proteins Mfn1 and OPA1 were decreased, while the levels of mitochondrial fission proteins Fis1, MFF and mitochondrial translocation of Drp1 were increased after IR‐783 exposure. (iii) Our in vivo study also confirmed that IR‐783 markedly increased the expression of Drp1 in mitochondria and the colocalization of Drp1 and mitoTracker. (iv) Knockdown of Drp1 significantly increased the average length of mitochondria and blocked mitochondrial fission mediated by IR‐783. (v) Knockdown of Drp1 also abrogated IR‐783‐induced loss of MMP, ATP depletion and mPTP opening, as well as apoptosis. To the best of our knowledge, this is the first report to demonstrate that Drp1‐mediated mitochondrial fission is required for IR‐783‐induced apoptosis in human breast cancer cells.

In summary, our findings demonstrated that Drp1‐mediated mitochondrial fission played an important role in IR‐783‐induced apoptosis in human breast cancer cells, and knockdown of Drp1 blocked mitochondrial fission, loss of MMP, ATP depletion, mPTP opening and apoptosis induced by IR‐783. Our findings suggest that Drp1 is a potent molecular target and IR‐783 might be an effective novel agent for clinical breast cancer treatment.

## CONFLICTS OF INTEREST

No potential conflict of interests were disclosed.

## AUTHORS’ CONTRIBUTIONS

Qin Tang, Wu‐Yi Liu, Guo‐Bing Li and Rong Zhang designed the research; Qin Tang, Wu‐Yi Liu, Qian Zhang, Jing‐Bin Huang, Min Zhou and Chang‐Peng Hu performed the research; Qin Tang, Qian Zhang, Ya‐Li Liu, Qing Wang, Fang‐Fang Sheng, Jing‐Bin Huang and Guo‐Bing Li analysed the data; Qin Tang, Wu‐Yi Liu, Guo‐Bing Li and Rong Zhang wrote the manuscript. All authors reviewed the manuscript.
