# Corrigendum

**DOI:** 10.1111/jcmm.17363

**Published:** 2022-06-06

In Qin Tang et al,^1^ the published article contains errors in Figure 4B. The correct figure is shown below. The authors state that this correction does not affect the other results of Figure 4, nor does it affect the conclusion of this article.

**FIGURE 4:**
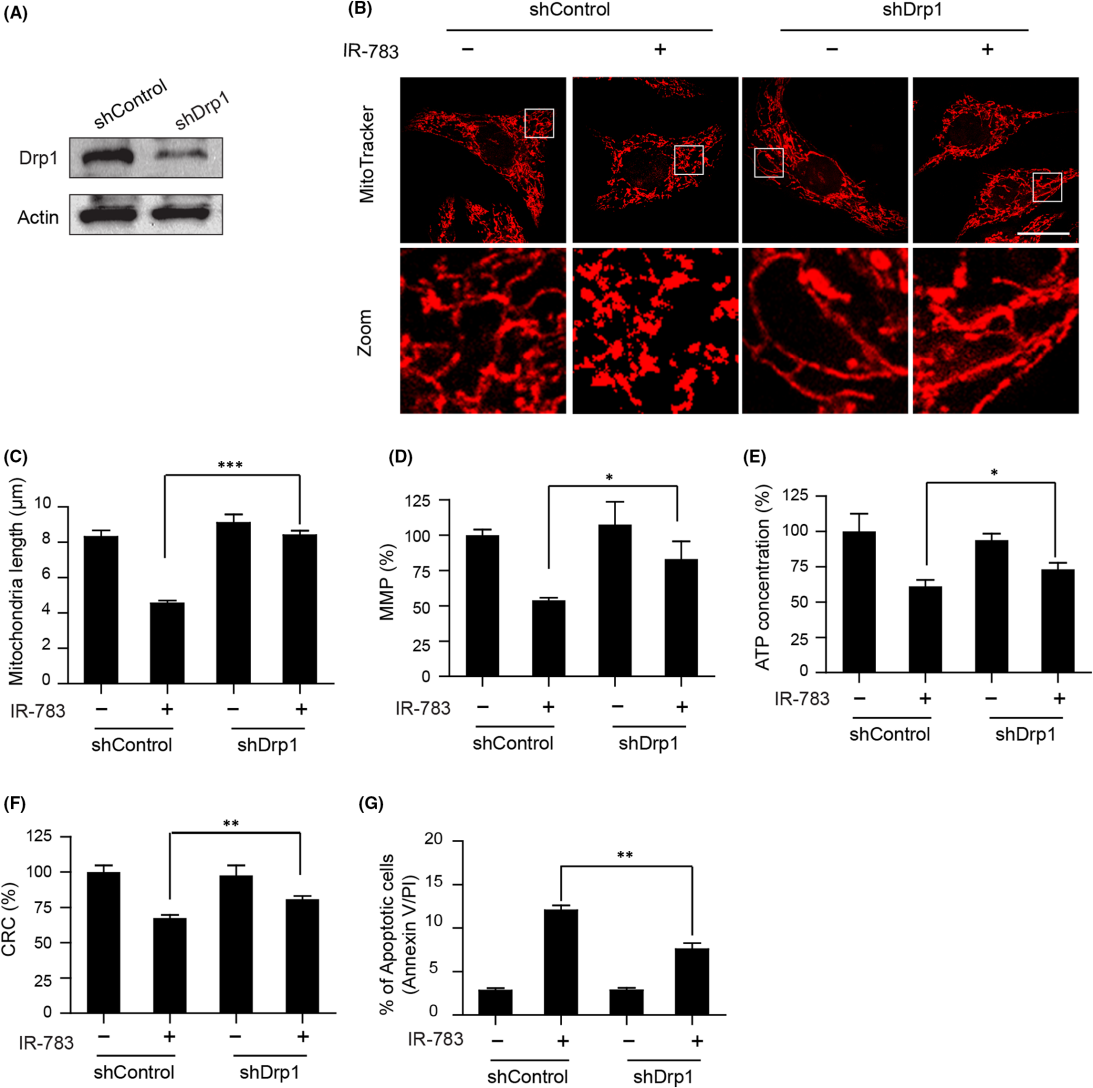
Knockdown of Drp1 blocked IR‐783‐induced mitochondrial fission, loss of MMP, ATP depletion, mPTP opening and apoptosis. (A) MDA‐MB‐231 cells stably expressing non‐target shRNA (shCon) or Drp1 shRNA (shDrp1) were lysed and analysed by western blot. Actin was used as the loading control. (B) shCon and shDrp1 cells were treated with or without 40 μM IR‐783 for 24 h. Mitochondria were then stained using MitoTracker Red CMXRos (red) and observed under a confocal microscope. Scale bars: 20 μm. (C) Average mitochondrial length was counted in 30 cells. (D) shCon and shDrp1 cells were treated with or without 40 μM IR‐783 for 24 h; then, the cells were stained with rhodamine‐123. The MMP was measured by fluorescence microplate. (E) Measurement of intracellular content of ATP by Luminometer Microplate reader. (F) Cells were stained with calcein‐AM+CoCl2 and tested by fluorescence microplate. The calcium retention capacity (CRC) contrast to control group is an index of the opening of mPTP. (G) Cells were stained with annexin V‐FITC/PE, and the percentage of apoptotic cells was measured by flow cytometry. The results were counted in three independent experiments *(*n = 3). Error bars represent the mean ±S.D. (*p < 0.05, **p < 0.01, ***p < 0.001)
