# Supplementary material for: Dynamin‐related protein 1‐mediated mitochondrial fission contributes to IR‐783‐induced apoptosis in human breast cancer cells
Source: J Cell Mol Med. 2018 Jul 11;22(9):4474–85. doi: 10.1111/jcmm.13749 (PMC6111821; doi:10.1111/jcmm.13749)
Supplement: Supplementary file 1 [file JCMM-22-4474-s001.docx]

**Supplementary materials**

**Dynamin-related protein 1-mediated mitochondrial** **fission contributes to** **IR-783 induced apoptosis in human breast cancer cells**

Qin Tang·Wuyi Liu·Qian Zhang·Jingbin Huang·Changpeng Hu·Yali Liu·Qing Wang·Min Zhou·Wenjing Lai·Fangfang Sheng·Guobing Li·Rong Zhang


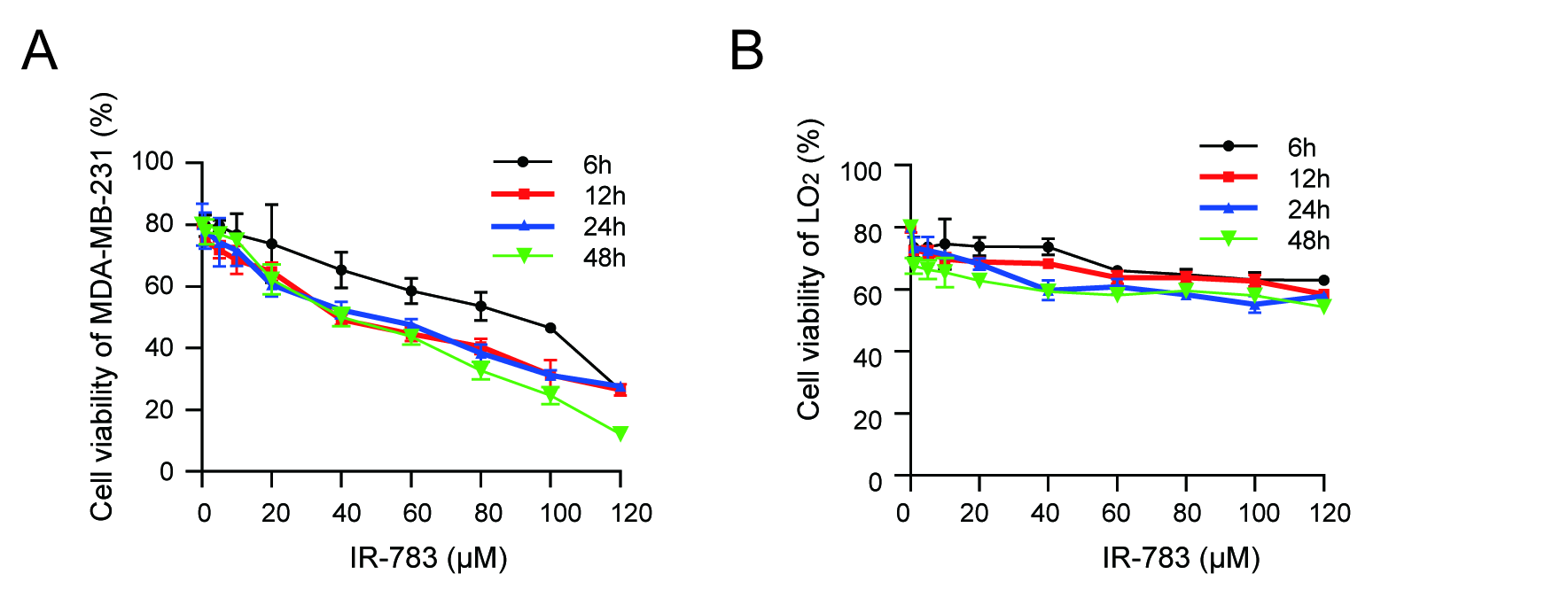


**Supplemental Figure 1: IR-783 selectively inhibits cell viability in human breast cancer cells but not normal hepatocytes.** Human breast cancer MDA-MB-231 cells (A) and human normal hepatocyte LO2 cells (B) were treated with IR-783 for different concentrations and different time intervals, the cell viability of cells were detected using MTT assays. The results were counted in three independent experiments (*n*=3). Data are expressed as a percentage of the control, which was set at 100%.
